# Review: UK medicines likely to be affected by the proposed European Medicines Agency’s guidelines on phthalates

**DOI:** 10.1186/s40360-015-0018-9

**Published:** 2015-06-13

**Authors:** Lisa Jamieson, William McCully

**Affiliations:** Enucleo Limited, Farnham, Surrey, UK; Tillotts Pharma UK Ltd., Larbourne Suite, 8 The Stables, Wellingore Hall, Wellingore, Lincoln, LN5 0HX UK

**Keywords:** Environmental exposure, Phthalic acids, Dibutyl Phthalate (DBP), Diethyl Phthalate (DEP), Polyvinylacetate Phthalate (PVAP), Excipients, Phthalate

## Abstract

**Background:**

Phthalates are excipients in drug formulations. However, concerns have been raised about the effects of particular phthalates on reproduction and development. As a result the EMA has introduced guidelines for permitted daily exposure (PDE) limits for certain phthalates. Therefore, the objective of this study was to identify UK licensed medicines that contain the relevant phthalates and determine if they fall within the recommended PDE.

**Methods:**

The eMC was used to identify which UK licensed medicines contain the phthalates in question. Companies were then contacted for information on the phthalate levels in their products, which was compared with the PDE recommended by the EMA.

**Results:**

The eMC search revealed that 54 medicines contained at least one of the phthalates in question. However, only six medicines, namely Asacol 800 mg MR (Warner Chilcott UK), Epilim 200 Gastro-resistant tablets (Sanofi), Prednisolone 2.5 mg and 5 mg Gastro-resistant tablets (Actavis UK), Vivotif (Crucell Italy S.r.l), and Zentiva 200 mg Gastro-resistant tablets (Winthrop Pharmaceuticals UK), were identified as containing levels that exceeded the recommended PDE.

**Conclusions:**

These findings indicate that very few UK licensed medicines will be affected by the proposed EMA guidelines. For those medicines identified as exceeding recommendations, these findings highlight the need to instigate a risk-benefit review.

## Background

Phthalates are synthetic chemical esters of phthalic acid, that are broadly divided into low molecular weight (LMW) phthalates, which include the likes of dibutyl phthalate (DBP), diethyl phthalate (DEP) and dimethyl phthalate (DMP); high molecular weight (HMW) phthalates, which encompass butylbenzyl phthalate (BBzP), di-2-ethylhexyl phthalate (DEHP), di-isodecyl phthalate (DiDP), di-isononyl phthalate (DiNP) and di-n-octyl phthalate (DnOP); and phthalate polymers, such as cellulose acetate phthalate (CAP), hydroxypropyl methylcellulose phthalate (HPMCP) and polyvinyl acetate phthalate (PVAP). They confer numerous properties, including those as a lubricant, a solvent, a softener and a plasticizer, which increases flexibility and durability. Consequently they were once widely found in a variety of consumer products, thus leading to ubiquitous daily exposure [1].

However, concerns have been raised regarding the effects of certain phthalates on reproduction and development. These worries predominantly stem from their endocrine-disrupting properties, and associated anti-androgen implications. They have been well documented pre-clinically, particularly in the rat, where prenatal exposure to particular phthalates has affected male and female offspring, with respect to numerous parameters including anogenital distance (AGD), gender ratio, nipple retention, ear and eye unfolding, vaginal opening and foetal weight and viability [2–4]. In addition, it has been demonstrated that their effects are additive when combined with each other, as well as, different classes of anti-androgen chemicals [5]. In fact the endocrine-disrupting effects of phthalates in the rat are so robust that within endocrinology laboratories, phthalates are often used as tools to induce testicular dysgenesis syndrome (TDS). By contrast to the preclinical arena, where there are vast studies evaluating the health implications of phthalates, too many to discuss within the scope of this article, clinical data are few and far between. Those that exist come from human association studies and suggest that prenatal exposure to certain phthalates reduces the AGD amongst male offspring, possibly indicative that it compromises virilisation [6, 7]. There is also evidence to suggest that prenatal exposure reduces masculine-play behaviour amongst pre-school males [8]. Furthermore, evaluation of phthalate exposure during adulthood demonstrates that it may contribute to both a reduction in the levels of circulating steroid hormones and sperm quality in males, as well as reduced fertility, in both males and females [9–12].

Whilst it is acknowledged that clinical data are limited and, in some cases inconsistent, regulatory bodies affiliated with consumer goods that contain phthalates deemed it necessary to take precautionary measures. Consequently, guidelines have been developed aimed at reducing exposure to certain phthalates in cosmetics [13], childcare articles [14], plastics in contact with food [15] and medical devices [16–18]. Certain medicines represent a source of phthalate exposure, where they exist as excipients, that is, inactive components. Since phthalates are insoluble in acidic environments and soluble in neutral and alkaline conditions, they are commonly used as plasticizing agents in gastro-resistant film coatings for tablets, capsules, beads and granules, thus enabling targeted delivery of active ingredient(s) to the more alkaline environment of the intestine. This is likely the reason that drugs for gastrointestinal indications have been identified as particularly high sources of phthalate exposure [19, 20]. Furthermore, animal and human pharmacokinetic studies have shown that LMW phthalates, such as DBP and DEP, have near complete intestinal absorption, with 78–90 % of the administered dose excreted in the urine within 24 h [21–23]. However, for the HMW phthalates CAP, PVAP and HPMCP, there is currently no pharmacokinetic data available.

Accordingly in 2012, the Food and Drug Administration (FDA) also developed guidelines aimed at minimising phthalate exposure in products regulated by the Centre for Drug Evaluation and Research (CDER) [24]. Specifically, the Agency determined that there is evidence that exposure to DBP and DEHP from pharmaceuticals presents a potential risk of developmental and reproductive toxicity. While the Agency recognised that drug products may carry inherent risks, it stated that DBP and DEHP are used as excipients, and safer alternatives are available. Therefore, the Agency recommends that DBP and DEHP be avoided as excipients in CDER-regulated drug and biologic products [24]. In line with the FDA, the EMA’s Committee for Medicinal Products for Human Use (CHMP) is currently drafting its own recommendations on the use of phthalates as excipients in human medicinal products [1]. Whilst these guidelines have yet to be finalised, they are expected to propose permitted daily exposures (PDE) of ≤ 0.01, 4 and 2 mg/kg for DBP, DEP and PVAP, respectively and are predicted to be enforced in 2015. For existing authorised medicinal products, the EMA is proposing to set a time limit of three years (after coming into force of the final guideline) for the implementation of formulation changes and consequential regulatory applications, as necessary. Ahead of their implementation, the authors of this study aimed to identify which United Kingdom (UK)- licensed drugs are likely to be affected by the proposed EMA guidelines, in order to help prepare for potential consequences.

## Methods

**Fig. 1:**
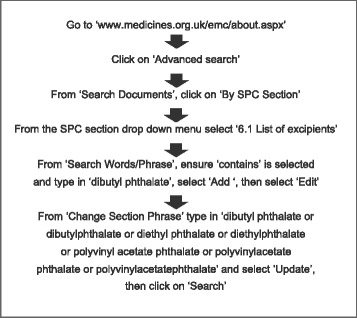
Algorithm for using the eMC to identify which UK licensed medicines contain at least one of the three precautionary phthalates, DBP, DEP and PVAP, named in the draft EMA guidelines on the use of phthalates as excipients in human medicinal products

The first step in this process was to identify which UK licensed medicines contain at least one of the three precautionary phthalates, DBP, DEP and PVAP, named in the draft EMA guidelines. The electronic Medicines Compendium (eMC) [25] was deemed an effective way to find these medicines, as it contains up to date information about most medicines licensed in the UK and is checked and approved by either the Medicines and Healthcare products Regulatory Agency (MHRA) or the EMA. The eMC was utilised via the algorithm depicted in Fig. 1.

The eMC provided the medical information email address associated with each medicine found to contain the precautionary phthalates. The next stage was to contact the companies affiliated with each of the medicines identified as containing the precautionary phthalates. In many cases, a single medical information address was affiliated with numerous products, thus it was only necessary to issue emails to 28 different medical information companies, requesting information about the maximum daily exposure of the relevant phthalate(s) in their product(s). Twenty-seven companies were contacted using the medical information email address provided by the eMC and one was contacted by an online enquiry form. The companies were made aware of the proposed EMA guidelines and their phthalate containing product(s) was identified together with a request for information on the maximum daily exposure of the affiliated phthalate(s).

## Results

The eMC search to identify UK medicines containing at least one of the three precautionary phthalates, DBP, DEP and PVAP, named in the draft EMA guidelines, produced 50 hits (information correct as of 9^th^ June 2014), one of which was deemed a false positive because the drug was listed as discontinued (Maxolon SR 15 mg Capsules). The remaining 49 hits, consisted of 54 branded medicines, five of which contained DBP alone, 17 contained DEP alone, 27 contained PVAP alone, four contained DEP in combination with PVAP and one contained DBP in combination with DEP (Table 1). It should be noted that this list will only contain those drugs registered on the eMC.

**Table 1:** UK licensed medicines that contain DBP, DEP and/or PVAP and respective maximum daily exposures relative to proposed EMA guidelines

*Trade Name (generic name)*	License holder	Phthalate	Phthalate content (mg) per tablet/capsule	Maximum licensed daily dose of drug	Phthalate (mg) at maximum daily dose	Chronic/Acute	Use in pregnancy
*Phthalate in tablet/capsule/liquid formulation*							
Asacol 800 mg MR Tablets (mesalazine)	Warner Chilcott UK Ltd	DBP	8.00^c^	4.8 mg	48.00	Chronic	Benefit>Risk
Coracten XL 30 mg (Nifedipine)	UCB Pharma Ltd	DBP	0.14	90 mg	0.42	Chronic	No
Coracten XL 60 mg (Nifedipine)	UCB Pharma Ltd	DBP	0.28	90 mg	0.28	Chronic	No
Occlusal (Salicylic acid)	Alliance Pharmaceuticals	DBP	N/A	RTD	RTD	Acute	Benefit>Risk
Timodine Cream (Nystatin, Dimeticone, Hydrocortisone & Benzalkonium Chloride)	Alliance Pharmaceuticals	DBP	N/A	RTD	RTD	Acute	No
Vivotif (Salmonella enterica serovar Typhi)	Crucell Italy S.r.l	DBP / DEP	8.00 / 8.00	1 tablet	8.00 / 8.00	Acute	Benefit>Risk
Kenzem 120 mg SR Capsules (Diltiazem hydrochloride)	Kent Pharmaceuticals	DEP	NR	480 mg	NR	Chronic	No
Kenzem 90 mg SR Capsules (Diltiazem hydrochloride)	Kent Pharmaceuticals	DEP	NR	480 mg	NR	Chronic	No
Kenzem 60 mg SR Capsules (Diltiazem hydrochloride)	Kent Pharmaceuticals	DEP	NR	480 mg	NR	Chronic	No
Omeprazole 40 mg Gastro-resistant Capsules, Hard (Omeprazole)	Accord Healthcare Ltd	DEP	0.15	120 mg	0.45	Chronic^d^	Yes
Omeprazole 20 mg Gastro-resistant Capsules, Hard (Omeprazole)	Accord Healthcare Ltd	DEP	0.15	120 mg	0.90	Chronic^d^	Yes
Reminyl XL 24 mg Prolonged Release Capsules (Galantamine)	Shire Pharmaceuticals Ltd	DEP	NR	24 mg	NR	Chronic	Benefit>Risk
Reminyl XL 16 mg Prolonged Release Capsules (Galantamine)	Shire Pharmaceuticals Ltd	DEP	NR	24 mg	NR	Chronic	Benefit>Risk
Reminyl XL 8 mg Prolonged Release Capsules (Galantamine)	Shire Pharmaceuticals Ltd	DEP	NR	24 mg	NR	Chronic	Benefit>Risk
Rheumatac Retard 75 mg Tablets (Diclofenac sodium)	Adipharm Mercury Company Ltd	DEP	0.95	150 mg	1.90	Acute	No
Surgical Spirit BP (Virgin castor oil & Methyl salicylate	Thornton & Ross Ltd	DEP	N/A	N/A	UTQ	Acute	Benefit>Risk
Videx EC 400 mg Gastro-resistant Capsules (Didanosine)	Bristol-Myers Squibb Pharmaceuticals Ltd	DEP	RTD	400 mg	RTD	Chronic	Benefit>Risk
Videx EC 250 mg Gastro-resistant Capsules (Didanosine)	Bristol-Myers Squibb Pharmaceuticals Ltd	DEP	RTD	400 mg	RTD	Chronic	Benefit>Risk
Videx EC 200 mg Gastro-resistant Capsules (Didanosine)	Bristol-Myers Squibb Pharmaceuticals Ltd	DEP	RTD	400 mg	RTD	Chronic	Benefit>Risk
Videx EC 125 mg Gastro-resistant Capsules (Didanosine)	Bristol-Myers Squibb Pharmaceuticals Ltd	DEP	RTD	400 mg	RTD	Chronic	Benefit>Risk
Volsaid Retard 100 mg Tablets (Diclofenac Sodium)	Chiesi Ltd	DEP	1.27	100 mg	1.27	Acute	No
Volsaid Retard 75 mg Tablets (Diclofenac Sodium)	Chiesi Ltd	DEP	0.95	150 mg	1.90	Acute	No
Boots Constipation Relief Tablets 40s (Bisacodyl)	Dr. Reddy’s Laboratories (UK) Ltd	DEP	NR	2 tablets	NR	Acute	No
Epilim 500 Gastro-resistant Tablets (Sodium valproate)	Sanofi	DEP / PVAP	2.31 / 23.31	2500 mg	11.55 / 116.55	Chronic	No
Epilim 200 Gastro-resistant Tablets (Sodium valproate)	Sanofi	DEP / PVAP	1.23 / 12.43	2500 mg	14.76 / 149.16	Chronic	No
Zentiva 500 mg Gastro-resistant Tablets (Sodium valproate)	Winthrop Pharmaceuticals UK Ltd	DEP / PVAP	2.31 / 23.31	2500 mg	11.55 / 116.55	Chronic	No
Zentiva 200 mg Gastro-resistant Tablets (Sodium valproate)	Winthrop Pharmaceuticals UK Ltd	DEP / PVAP	1.23 / 12.43	2500 mg	14.76 / 149.16	Chronic	No
Boots Alternatives Laxative Tablets (Senna, Aloin, Cascara bark extract)	G.R. Lane Health Products Ltd	PVAP	2.10	2 tablets	4.20	Acute	No
Boots Period Pain Relief 250 mg Gastro-resistant Tablets (Naproxen)	Teva UK Ltd	PVAP	NR	500 mg	NR	Acute	No
Deltacortril 2.5 mg Gastro-resistant Tablets (Prednisolone)	Alliance Pharmaceuticals	PVAP	RTD	60 mg	RTD	Chronic^d^	Benefit>Risk
Deltacortril 5 mg Gastro-resistant Tablets (Prednisolone)	Alliance Pharmaceuticals	PVAP	RTD	60 mg	RTD	Chronic^d^	Benefit>Risk
Disipal 50 mg Tablets (Orphenadrine hydrochloride)	Astellas Pharma Ltd	PVAP	17.30	400 mg	138.40	Chronic	Benefit>Risk
Feminax Ultra 250 mg Gastro-resistant Tablets (Naproxen)	Teva UK Ltd	PVAP	NR	750 mg	NR	Acute	No
Ferrous Gluconate 300 mg Tablets (Ferrous gluconate)	Kent Pharmaceuticals	PVAP	NR	1800 mg	NR	Chronic^d^	Yes
Nardil 15 mg Tablets (Phenelzine)	Archimedes Pharma UK Ltd	PVAP	1.42	90 mg	8.52	Chronic	No
Prednisolone 5 mg Gastro-resistant Tablets (Prednisolone)	Actavis UK Ltd	PVAP	12.00	60 mg	144.00	Chronic^d^	Benefit>Risk
Prednisolone 2.5 mg Gastro-resistant Tablets (Prednisolone)	Actavis UK Ltd	PVAP	12.00	60 mg	288.00	Chronic^d^	Benefit>Risk
Pancrex Granules (Pancreatin)	Essential Pharmaceuticals Ltd	PVAP	RTD	variable^a^	RTD	Chronic	Benefit>Risk
Pancrex V Tablets (Pancreatin)	Essential Pharmaceuticals Ltd	PVAP	RTD	variable^a^	RTD	Chronic	Benefit>Risk
Pancrex V Forte Tablets (Pancreatin)	Essential Pharmaceuticals Ltd	PVAP	RTD	variable^a^	RTD	Chronic	Benefit>Risk
*Phthalate in tablet/capsule logo ink*							
Aloxi 500 μg Soft Capsules (Palonosetron)	Sinclair IS Pharma	PVAP	UTQ	500 μg	UTQ	Acute	No
Amitiza 24 μg Soft Capsules (Lubiprostone)	Sucampo Pharma Europe Ltd	PVAP	0.21	48 μg	0.42	Acute	No
Anadin Ultra Double Strength/LiquiFast 400 mg Capsules (Aspirin)	Pfizer Consumer Healthcare	PVAP	<0.01	1200 mg	0.03	Acute	No
Anadin Ultra/LiquiFast 200 mg Capsules (Aspirin)	Pfizer Consumer Healthcare	PVAP	<0.01	1200 mg	0.05	Acute	No
Aptivus 250 mg soft Capsules (Tipranavir)	Boehringer Ingelheim Ltd	PVAP	UTQ	1000 mg	UTQ	Chronic	Benefit>Risk
Benadryl Allergy Liquid Release 10 mg Capsules (Certirizine dihydrochloride)	McNeil Products Ltd	PVAP	1.00	10 mg	1	Chronic^d^	Benefit>Risk
Nurofen Express 200 mg Liquid Capsules (Ibuprofen)	Reckitt Benckiser Healthcare (UK) Ltd	PVAP	NR	1200 mg	NR	Acute	No
Nurofen Express 400 mg Liquid Capsules (Ibuprofen)	Reckitt Benckiser Healthcare (UK) Ltd	PVAP	NR	1200 mg	NR	Acute	No
Targretin 75 mg Capsules (Bexarotene)	Eisai Ltd	PVAP	UTQ	21 capsules^b^	UTQ	Acute	No
Xtandi 40 mg Soft Capsules (Enzalutamide)	Astellas Pharma Ltd	PVAP	UTQ	160 mg	UTQ	Acute	No
Zemplar Soft Capsules 2 μg (Paricalcitol)	AbbVie Ltd	PVAP	0.86	32 μg	13.76	Chronic	Benefit>Risk
Zemplar Soft Capsules 1 μg (Paricalcitol)	AbbVie Ltd	PVAP	0.86	32 μg	27.52	Chronic	Benefit>Risk

All calculations are based on the maximum licensed dose. If a drug has multiple indications, the indication with the highest dose was used for the calculation. For drugs that cannot be given at the maximum dose due to their dose increment, (i.e. sodium valproate 200 mg – max dose 2500 mg), the maximum achievable dose within the product license was used

*UTQ* denotes unable to quantify, *RTD* denotes licence holder refused to declare, *NR* denotes no response

^a^ Dosing regime of Pancrex is dependent on frequency of meals/snacks

^b^ Based on a dose of 650 mg/m^2^/day for a person with a body surface area of 2.38–2.62 m^2^

^c^ Information in the public domain [26, 27], license holder refused to confirm

^d^ These medications are also used in acute settings

Whilst some companies provided maximum daily exposure as requested, thus enabling direct comparison with those set out in the draft guidelines, others did not specify exposure levels but instead commented on how exposure compared with the guidelines. In some instances, companies stipulated the amount of phthalate present in a single unit of the medicine, thus requiring the authors to calculate the maximum daily exposure based on maximum indicated dose as per the ‘summary of product characteristics’ (SPC). There were five cases where companies stated that they were unable to quantify (UTQ) exposure and for 11 products, companies refused to declare (RTD). Remaining companies provided no response (NR) at all which amounted to 12 products. Responses are summarised in Table 1, however, we were unable to include the phthalate content for 23 products because either the licence holder refused to declare or provided no response.

The proposed permitted daily exposures (PDE) in the EMA guidance equates to < 0.7 mg for DBP, 280 mg for DEP and 140 mg for PVAP, for an individual with 70 kg body weight.

### Phthalate in tablet/capsule logo ink

For some preparations, the phthalate was contained in the tablet/capsule logo ink on the surface of the dosage formulation. In all of these preparations, the phthalate was PVAP. For those companies who responded, the level of PVAP in the ink in each preparation was below proposed EMA PDE in all products. In some cases, the manufacturer declared that the phthalate content was so low per dosage form that it was negligible or too low to measure accurately.

### Phthalate in tablet/capsule/liquid formulation

Where the phthalate was contained as an excipient within the dosage form, the level of phthalate varied considerably. The EMA proposes that the presence in medicinal products of DBP, DEP or PVAP at levels giving rise to daily exposures above the PDEs may be accepted as exceptions, on a case-by-case basis taking into consideration the intended patient population, the disease seriousness and the presence (or not) of alternative treatment options. Furthermore, the EMA also proposes that in severe or terminal disease conditions, the strict application of the PDE may not be considered necessary for DBP, DEP or PVAP-containing medicinal products, where the risk of reproductive and developmental toxicity is outweighed by the benefits of treatment for patients.

Consequently, the drugs were assessed in terms of whether or not they are used acutely or chronically. Where a drug may be used in either an acute or a chronic condition, it was categorised as “chronic^d^”, due to the possibility that the drug may be prescribed on a long-term basis. Drugs were also categorised according to whether or not they are licensed for use in pregnancy. The SPC for some drugs advises caution in pregnancy or the drug may be used where the benefit outweighs the risk. For both of these categories, drugs were described as “benefit > risk” (Table 1).

Where the licence holder supplied information regarding DBP content, the level was above the proposed EMA PDE of 0.7 mg for 70 kg body weight in Asacol 800 mg MR (Warner Chilcott UK Ltd) and Vivotif (Crucell Italy S.r.l), the levels being 48 mg and 8 mg, respectively, at the maximum daily dose.

For DEP, the proposed EMA PDE is 280 mg per 70 kg body weight. Where the licence holder supplied information, this level was not exceeded at maximum daily dose in the DEP-containing products.

The proposed EMA PDE for PVAP is 140 mg per 70 kg body weight. There were two PVAP-containing products where the level exceeded this. These were Prednisolone 2.5 mg Gastro-resistant Tablets and Prednisolone 5 mg Gastro-resistant Tablets (Actavis UK Ltd), where the levels at the maximum licensed daily dose were 288 and 144 mg, respectively.

Four preparations contained a combination of DEP and PVAP. Of these, Epilim 200 Gastro-resistant Tablets (Sanofi) and Zentiva 200 mg Gastro-resistant Tablets (Winthrop Pharmaceuticals UK Limited) contained a level of PVAP that was above the EMA PDE. The PVAP level was 149.16 mg in both sodium valproate 200 mg formulations.

## Discussion

### Summary

The aim of this study was to identify which UK licensed medicines are likely to be affected by proposed EMA guidelines on the use of phthalates as excipients in human medicinal products. Although we attempted to identify as many phthalate-containing preparations as possible by reviewing SPCs, this list cannot be considered exhaustive. For 23 products, the licence holder refused to declare the phthalate content or gave no response. At face value, it appeared that many medicines would be impacted by the recommendations as 54 medicines were identified as containing the precautionary phthalates DBP, DEP and PVAP, named in the guidelines. However, for those medicines where companies responded, once maximum daily phthalate exposures were established, only six branded medicines, namely Asacol 800 mg MR (Warner Chilcott UK Ltd), Epilim 200 Gastro-resistant Tablets (Sanofi), Prednisolone 2.5 and 5 mg Gastro-resistant Tablets (Actavis UK Ltd), Vivotif (Crucell Italy S.r.l), and Zentiva 200 mg Gastro-resistant Tablets (Winthrop Pharmaceuticals UK Limited), were identified as exceeding the EMA’s proposed recommendations. Thus, this study will help to appease those concerned about the implications of enforcement of these guidelines.

### Strengths and limitations

To the authors’ knowledge, this study has provided the first review of the presence of phthalates in UK licenced medications. Furthermore, it has identified, where possible, which phthalate-containing medications will be affected by EMA guidance once it comes into practice. By virtue of the limited information in the public domain and the proprietary nature of drug formulations, information on the concentration of phthalates was limited to only 57 % of the drugs identified. This highlights the potential difficulty in clinical practice when undertaking a risk/benefit approach in the preceding 3 years before the enforcement of this guidance. In addition, not all SPC’s are available on the eMC, further hindering the ability of making an informed decision in certain patient populations.

## Conclusion

For those medicines identified as exceeding the EMA’s recommendations, this study has highlighted the need to instigate a risk-benefit review, particularly in patients of childbearing age and/or with chronic conditions. To facilitate this process, the EMA, suggests taking into account factors such as the presence of non-phthalate containing alternatives, the intended patient population and the severity of the disease being treated.

## Abbreviations

AGD: Anogenital distance
BBzP: Butylbenzyl phthalate
CAP: Cellulose acetate phthalate
CDER: Centre for Drug Evaluation and Research
DBP: Dibutyl phthalate
DEHP: Di-2-ethylhexyl phthalate
DEP: Diethyl phthalate
DiDP: Di-isodecyl phthalate
DMP: Dimethyl phthalate
DnOP: Di-n-octyl phthalate
EMA: European Medicines Agency
eMC: Electronic medicines compendium
FDA: Food and Drug Administration
HMW: High molecular weight
HPMCP: Hydroxypropyl methylcellulose phthalate
LMW: Low molecular weight
MHRA: Medicines and Healthcare products Regulatory Agency
NR: No response
PDE: Permitted daily exposure
PVAP: Polyvinylacetate phthalate
RTD: Refused to declare
SPC: Summary of product characteristics
TDS: Testicular dysgenesis syndrome
UTQ: Unable to quantify
